# Supplementary material for: Ezh2 phosphorylation state determines its capacity to maintain CD8+ T memory precursors for antitumor immunity
Source: Nat Commun. 2017 Dec 14;8:2125. doi: 10.1038/s41467-017-02187-8 (PMC5730609; doi:10.1038/s41467-017-02187-8)
Supplement: Supplementary file 4 — Supplementary Information [file 41467_2017_2187_MOESM4_ESM.pdf]

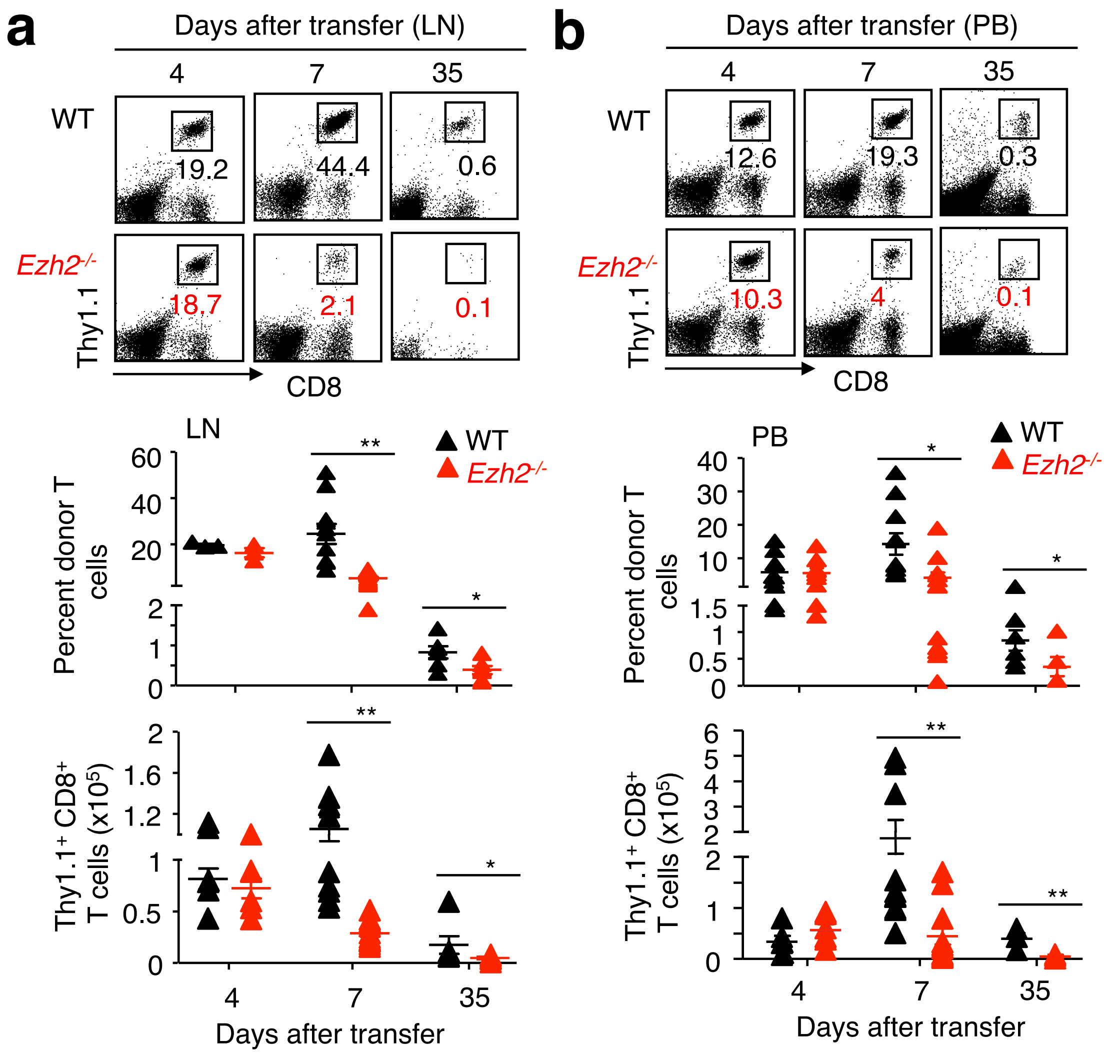

Supplementary Fig. 1. Ezh2 promotes CD8$^+$ T cell anti-tumor immunity and memory formation. WT and *Ezh2*$^{-/-}$ T_N Pmel-1 cells (1x10^6, Thy1.1$^+$) were transferred into sub-lethally irradiated non-tumor-bearing B6 mice, followed by immunization with IL-2 and gp100-DCs for 3d. Donor T cells were collected from the LN (**a**) and circulating peripheral blood (PB) (**b**) 4d, 7d and 35d after adoptive transfer. Plots and graphs show the frequency and numbers of donor T cells. Plots and graphs show the percentage of WT and *Ezh2*$^{-/-}$ Pmel-1 cells. *: $p<0.05$, and **: $p<0.01$ (two-tailed unpaired t-test). Data are representatives of two independent experiments (n=3-9 mice per group in each, mean ± SD).

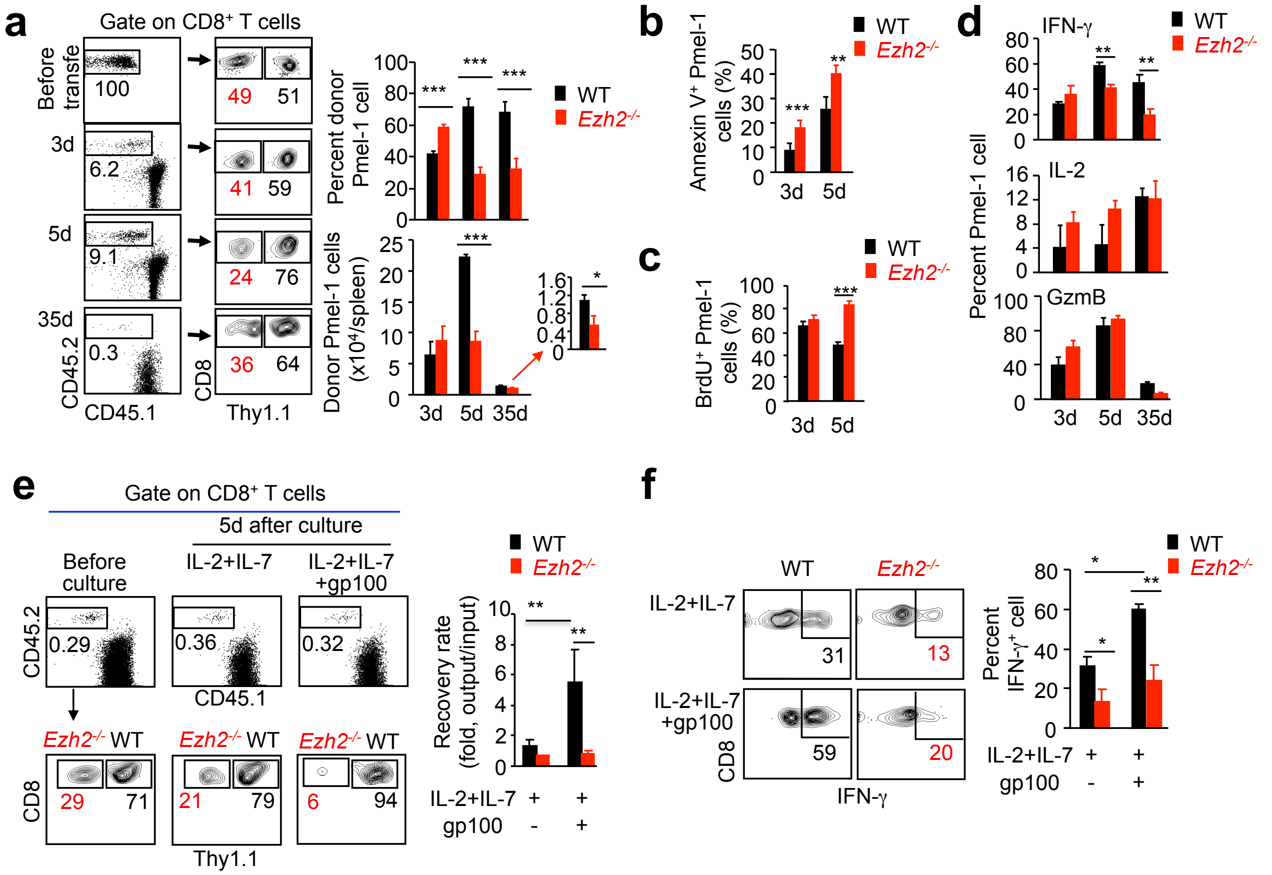

Supplementary Fig. 2. Ezh2 is required for CD8$^+$ T cells to form functionally intact memory cells in lymphoreplete mice infected VVA-gp100 infection. WT naïve Pmel-1 cells (Thy1.1$^+$CD45.2$^+$, 5x10^4) and Ezh2$^{-/-}$ naïve Pmel-1 cells (Thy1.1$^-$CD45.2$^+$, 5x10^4) were co-transferred into B6/SJL (CD45.1$^+$) mice, followed by infection with VVA-gp100 (1.25x10^6 PFU). Donor cells were recovered from the spleen at the indicated time points after infection. (**a**) Plots and graphs show the percentage of fraction of WT and Ezh2$^{-/-}$ T cells in the spleen. (**b**) Graphs show the percentage of Annexin-V$^+$ cells within WT and Ezh2$^{-/-}$ T cell population. (**c**) BrdU was administered to the recipient mice 16h before analysis. Graphs show the percentage of donor T cells with incorporated BrdU. (**d**) Percentage of IFN-γ-, IL-2-, and GzmB-expressing cells after gating on donor WT (Thy1.1$^+$CD45.2$^+$) and *Ezh2*$^{-/-}$ (Thy1.1$^-$CD45.2$^+$) CD8$^+$ T cells. (**e-f**) Spleen mononuclear cells were recovered 35d after adoptive transfer, cultured *ex vivo* for additional 5d in the presence of IL-2+IL-7, with or without addition of gp100. (**e**) Plots and graphs show the percentage of donor CD8$^+$ T cells (upper) and the percent of donor WT (Thy1.1$^+$) and *Ezh2*$^{-/-}$ (Thy1.1$^-$) cells after gating on donor CD8$^+$ T cells (C45.2$^+$). Graph shows the recovery rate of WT and *Ezh2*$^{-/-}$ T cell number after and before culture. (**f**) IFN-γ-producing Pmel-1 cells were measured using flow cytometric analysis. *: $p<0.05$, **: $p<0.01$, and ***: $p<0.01$ (two-tailed unpaired t-test). Data are representative of two independent experiments with n=3 mice per group in each experiment (mean ± SD).

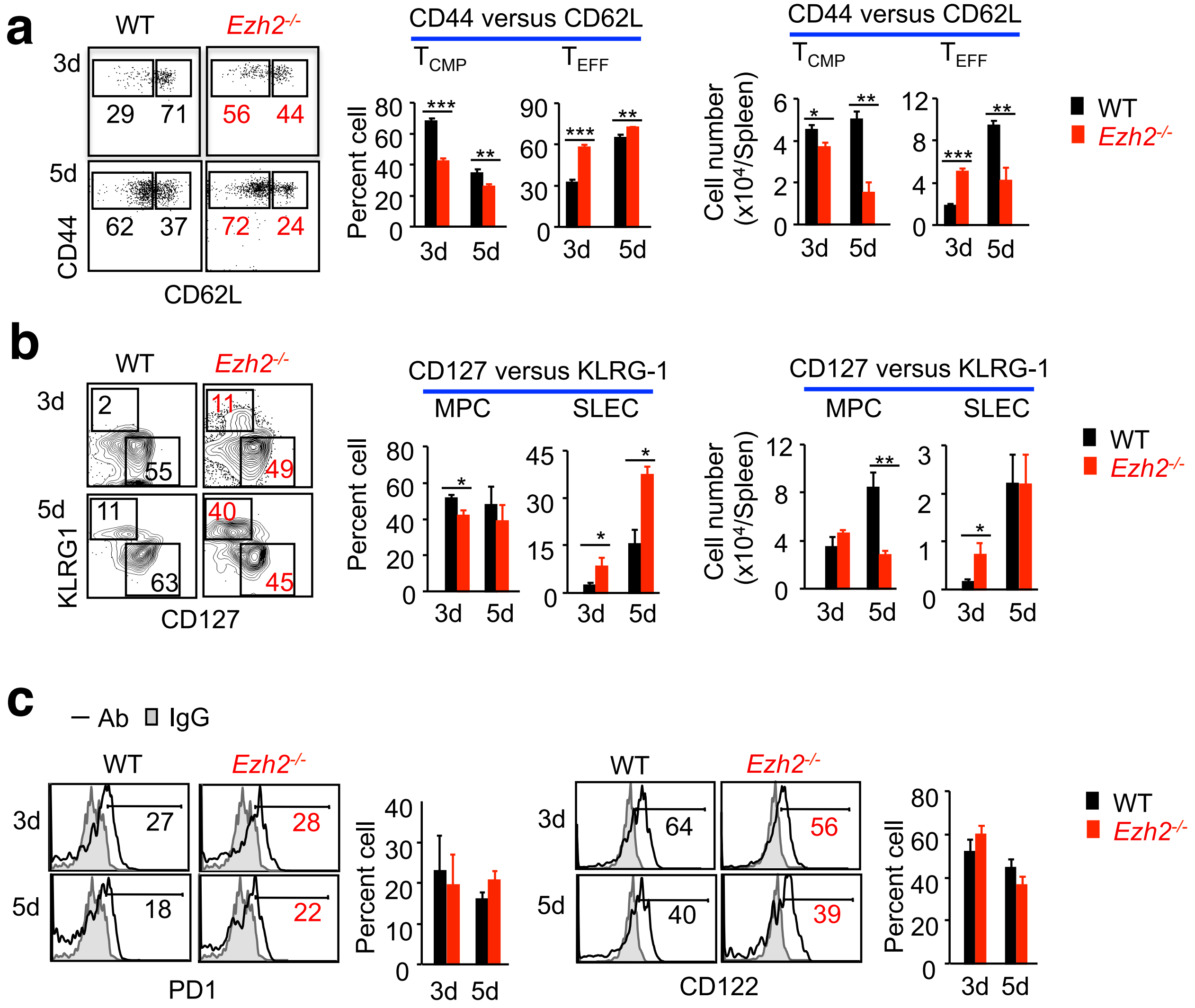

Supplementary Fig. 3. Ezh2 inhibition promotes terminal differentiation at the expense of T_{CMP}. WT naïve Pmel-1 cells (Thy1.1$^+$CD45.2$^+$, 5x10^4) and *Ezh2*$^{-/-}$ naïve Pmel-1 cells (Thy1.1$^-$ CD45.2$^+$, 5x10^4) were co-transferred into B6/SJL (CD45.1$^+$) mice, followed by infection with VVA-gp100 (1.25x10^6 PFU). Donor T cells were recovered from the spleen at 3d and 5d after infection to measure the presence of different subsets of CD8$^+$ T cells. (**a**) Plots and graphs show the percentage and number of T_{CMP} and T_{EFF}. (**b**) Percentage and number of MPC and SLEC assessed by staining with KLRG-1 by CD127. (**c**) Histograms and graphs show the expression of PD1 and CD122 on the surface of WT and *Ezh2*$^{-/-}$ CD8$^+$ T cells. *: p<0.05, **: p<0.01, and ***: p<0.001 (two-tailed unpaired t-test). Data are representative of two independent experiments with n=3 mice per group in each experiment (mean ± SD).

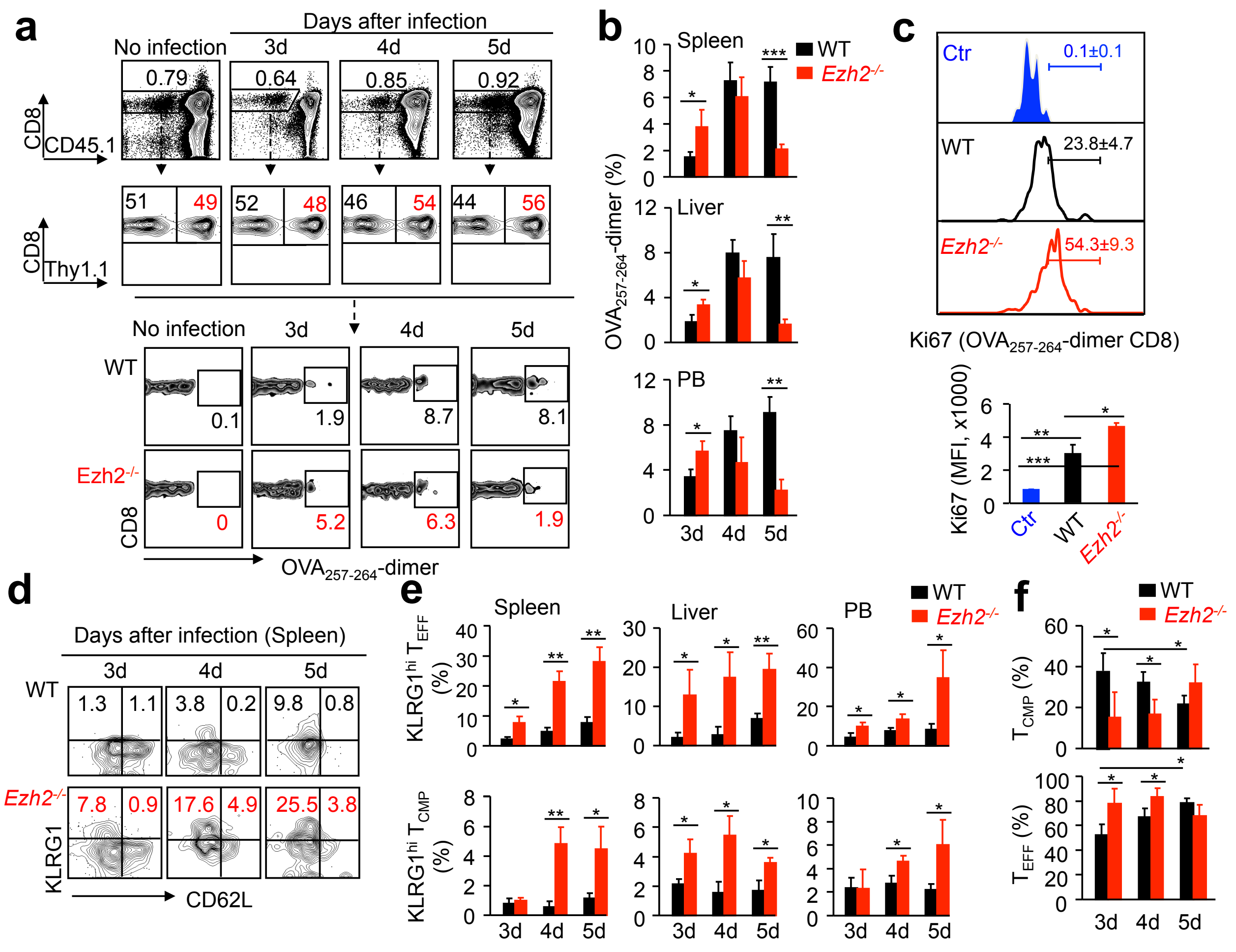

Supplementary Fig. 4. Ezh2 deficiency leads to preferential loss of endogenous memory precursors. Equal amount of WT (Thy1.1$^+$CD45.2$^+$) and *Ezh2*$^{-/-}$ (Thy1.1$^-$CD45.2$^+$) splenocytes (2x10^7) were co-transferred into lymphoreplete B6/SJL mice (Thy1.1$^-$CD45.1$^+$, n=3 for each group), followed by infection with VVA-OVA. At indicated days after infection, donor T cells were isolated, stained with the dimer binding to OT-I specific CD8$^+$ T cells. (**a**) Dot plots show the fraction of donor T cells in the spleen from mice at different days of infection. (**b**) Graphs show the percentage of donor OVA$_{257\text{-}264}$ dimer-specific CD8$^+$ T cells in spleen, liver and PB. (**c**) Histograms and graphs show the expression of Ki67 in donor-derived OVA$_{257\text{-}264}$ dimer-specific CD8$^+$ T cells. (**d, e**) Dot plots and graphs show the frequency of KLRG1^{hi} cells among donor OT-I-specific CD8$^+$ T cells. (**f**) Graphs show the fraction of T_{CMP} and T_{EFF} among donor OVA$_{257\text{-}264}$ dimer-specific CD8$^+$ T cells. Data (mean ± SD) are representatives of two independent experiments. *: p<0.05, **: p<0.01, and ***: p<0.001.

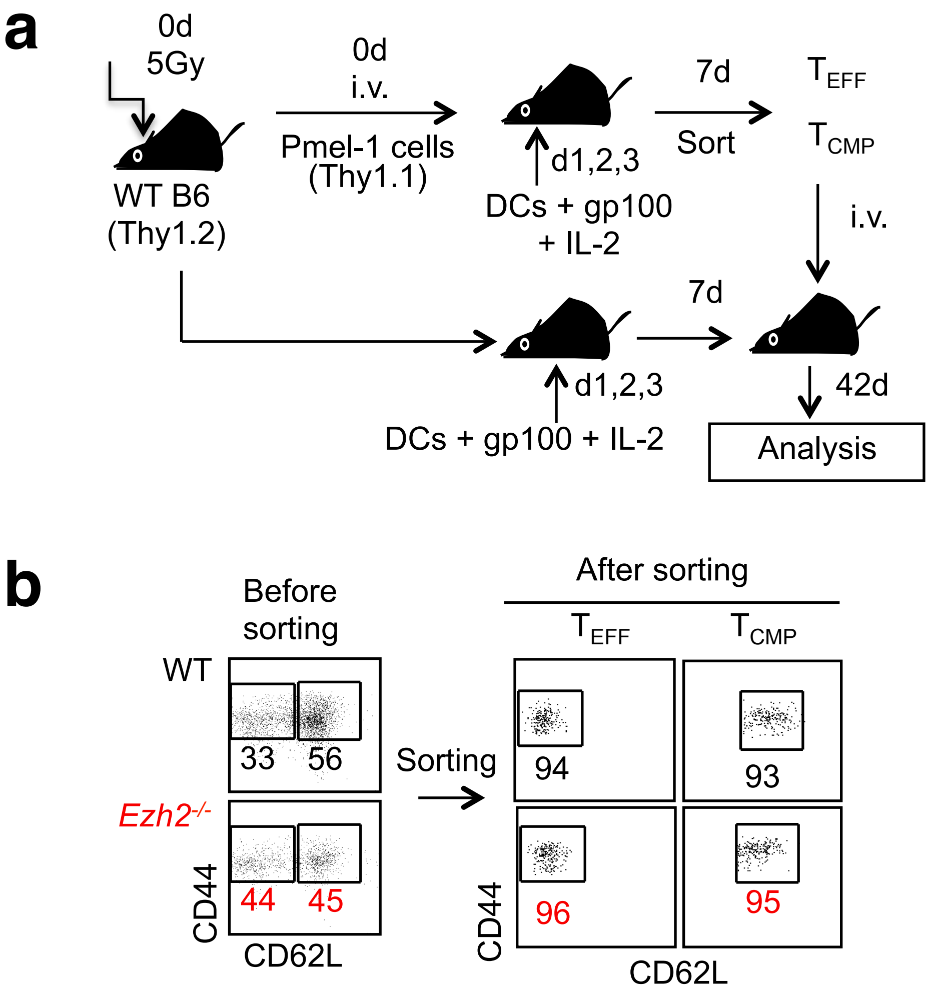

Supplementary Fig. 5. Purification and transfer of precursor memory T cells. (a) Schematic diagram of assessing the long-term impact of Ezh2 deficiency on the transition of memory precursor cells into mature memory T cells. WT and *Ezh2*$^{-/-}$ naïve Pmel-1 cells ($1x10^6$, Thy1.1$^+$) were transferred into sub-lethally irradiated B6 mice (Thy1.2$^+$), followed by treatment with IL-2 and gp100-DCs. T_{CMP} and T_{EFF} were highly purified using cell sorter from the spleen of these primary recipients 7d after transfer and transferred into immunization-matched secondary lymphodepleted B6 mice. Forty-two d later, donor cells were isolated from the spleen of these secondary recipients to measure their memory cell properties. (**b**) Dot plots show the fraction of WT and *Ezh2*$^{-/-}$ T_{CMP} and T_{EFF} before and after FACS sorting T cells pooled from 4 to 6 mice in each group.

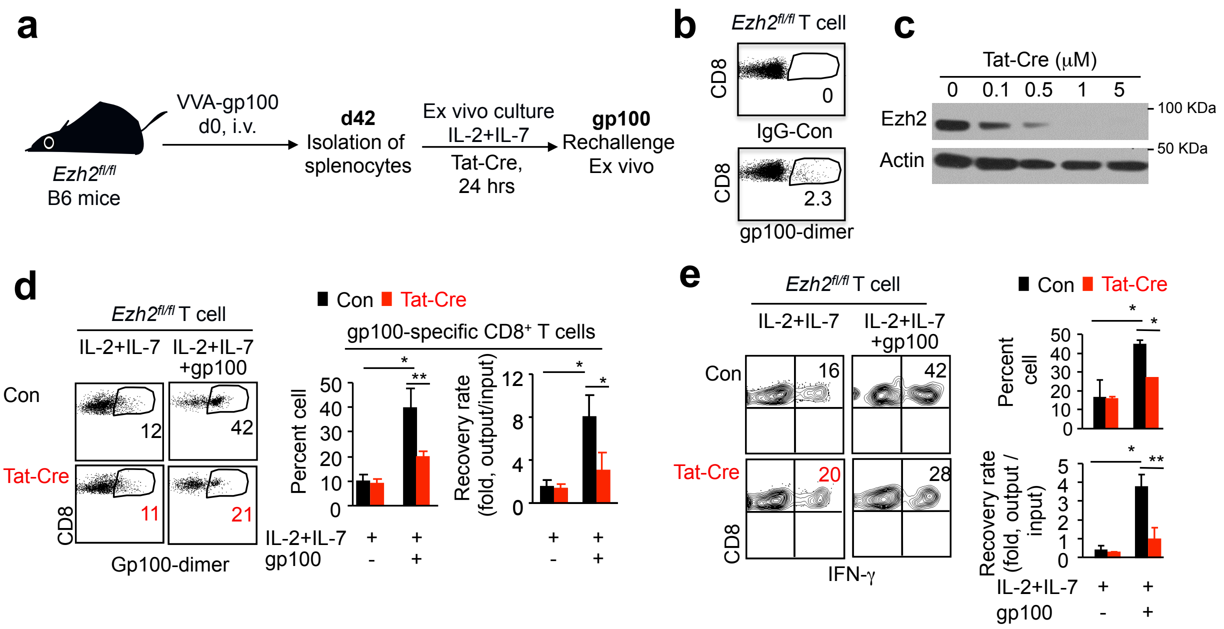

Supplementary Fig. 6. Selective deletion of Ezh2 in mature memory CD8+ T cells impairs their memory recall responses. *Ezh2fl/fl* B6 mice were infected with VVA-gp100 ($1.25x10^6$ PFU). Splenocytes were recovered at 42d after infection, cultured in the presence of IL-7(5ng/ml) + IL-15 (5ng/ml) and treated with or without TAT-Cre for 24hrs. After extensive wash with PBS, cells were re-cultured *ex vivo* for additional 5d in the presence of IL-2 + IL-7 with or without gp100. $H2D^b$-gp100 specific dimer was used to stain gp100-specific $CD8^+$ T cells. **(a) Schematic experimental design.** (**b**) Flow cytometric analysis shows gp100-specific $CD8^+$ T cells in the spleen 42d after immunization. (**c**) Immunoblots of *in vivo* recovered $CD8^+$ cells that were treated with various concentration of TAT-Cre. (**d**) Plots and graphs show the percentage and recovery rate of gp100-specific $CD8^+$ T cells in the culture with or without Ezh2 deletion by TAT-Cre. (**e**) IFN-γ production by gp100-specific $CD8^+$ T cells upon *ex vivo* deletion of Ezh2 and restimulation with gp100. *: $p<0.05$, and **: $p<0.01$ (two-tailed unpaired t-test). Data are representative of two independent experiments with n=3 mice per group in each experiment (mean ± SD).

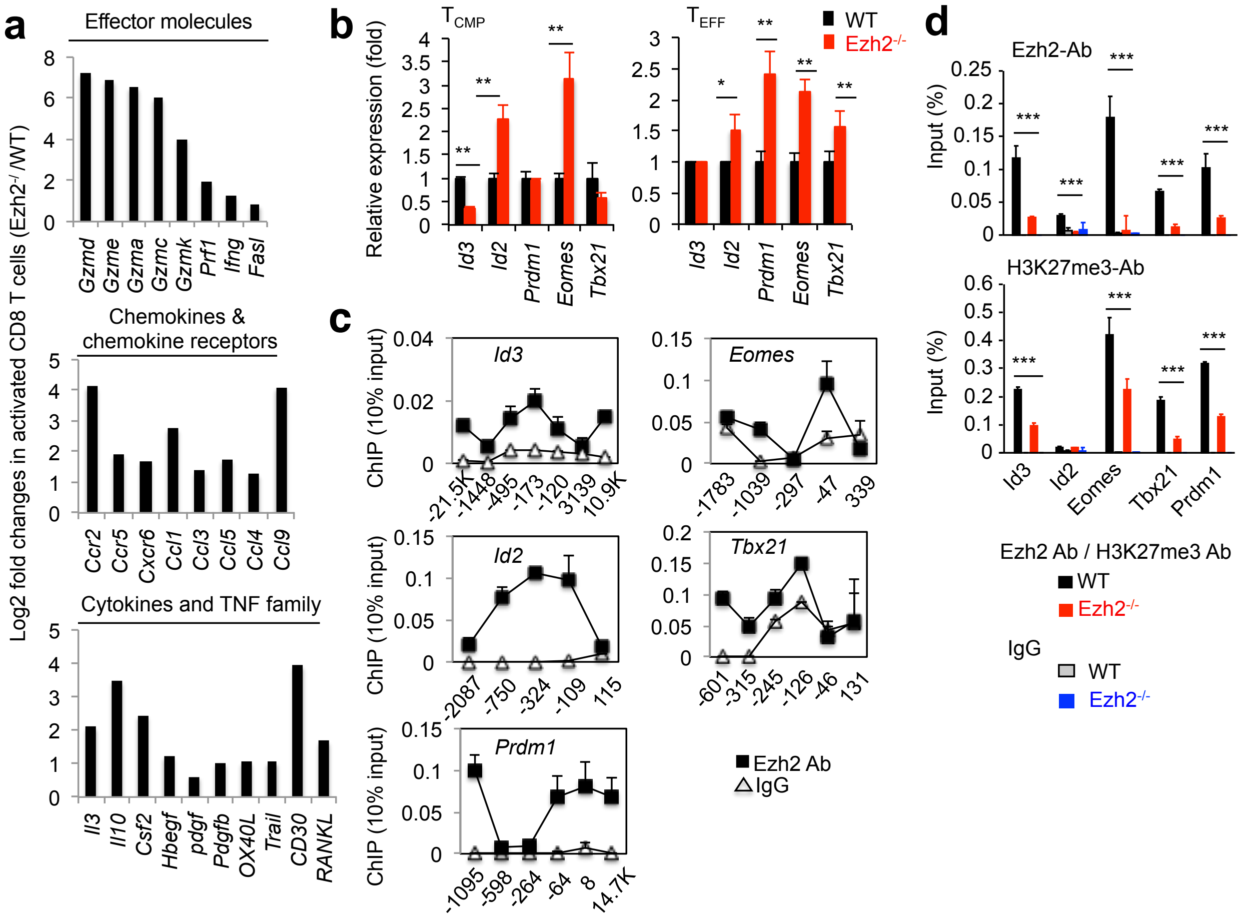

Supplementary Fig. 7. RNA-sequence profiling analysis identifies Ezh2-targeted genes in activated CD8^{+} T cells. WT and *Ezh2$^{-/-}$* naïve Pmel-1 cells were cultured in the presence of anti-CD3/CD28 Abs and IL-2. Three days later, cells were collected to extract RNA and chromatin. RNA sequencing was performed using freshly isolated WT T_N, *Ezh2$^{-/-}$* T_N, activated WT cells, and activated *Ezh2$^{-/-}$* cells. Using one-way ANOVA analysis, we selected transcripts with p<0.01 and q<0.01 for comparing paired groups and at least a 1.5-fold difference from the means for the paired groups. (**a**) Graphs showed the change of selected transcripts for comparing paired groups by RNA-seq profiling analysis. (**b**) WT and *Ezh2$^{-/-}$* naïve Pmel-1 cells (Thy1.1^{+}) were transferred into sublethally irradiated B6 mice (Thy1.2^{+}, n=3 for each group), followed by treatment with IL-2 and gp100/DCs for 3d as described in Fig.1b. At 7d of transfer, T_{CMP} and T_{EFF} were highly purified using FACS sorter to measure their expression of major TFs using real-time RT-PCR. (**c-d**) WT and *Ezh2$^{-/-}$* naive Pmel-1 cells were cultured in the presence of anti-CD3/CD28 Abs and IL-2. Cells were collected 3d later for ChIP analysis. (**c**) ChIP analysis of the deposition of Ezh2 or IgG in WT T cells at the different regions of these major TF loci. (**d**) ChIP analysis of the deposition of Ezh2, H3K27me3 or IgG within WT and *Ezh2$^{-/-}$* T cells at the promoter region of these major TF loci. *: p<0.05, **: p<0.01, and ***: p<0.001 (two tailed unpaired t-test). Data are representative of three independent experiments (**b-d**; mean ± SD).

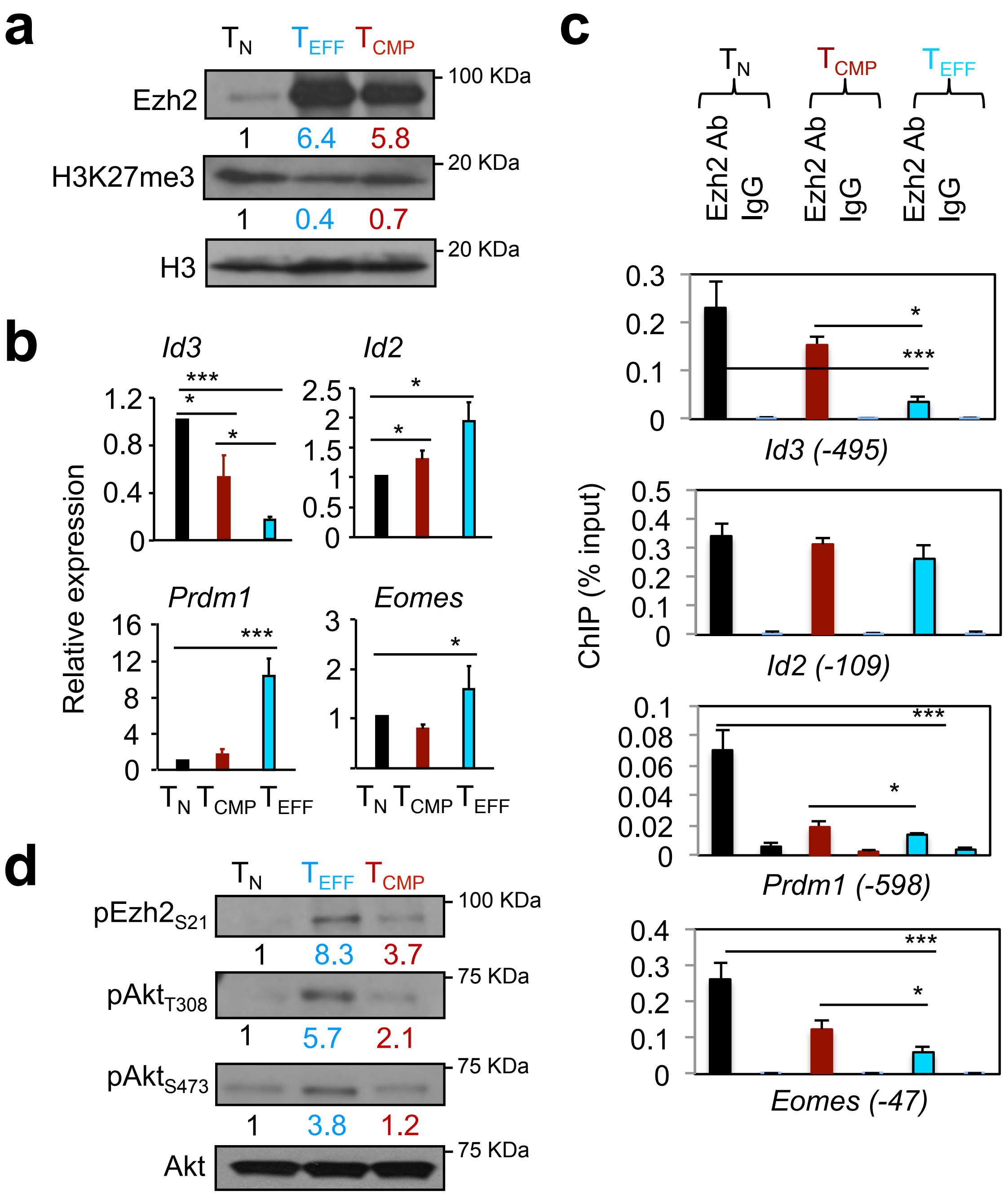

Supplementary Fig. 8. Akt-mediated phosphorylation of Ezh2 reduces Ezh2 function in activated CD8$^+$ T cells. WT and *Ezh2*$^{-/-}$ naïve Pmel-1 cells ($1x10^6$, Thy1.1$^+$) were transferred into sublethally irradiated B6 mice (Thy1.2$^+$), followed by treatment with IL-2 and gp100-DCs. T_{CMP} and T_{EFF} were highly purified using cell sorter from the spleen of these primary recipients 7d after transfer. (**a**) Immunoblots using Abs against Ezh2, H3K27me3 and H3. (**b**) Real-time PCR analysis of major TF transcripts. (**c**) ChIP analysis of Ezh2 binding to the promoter regions of these major TF loci. (**d**) Immunoblots of the expression of indicated proteins. *: p<0.05, and ***: p<0.001 (two-tailed unpaired t-test). Data are representative of three independent experiments (**a, d**), or two experiments with T cells pooled from four mice in each (**b,c**, mean ± SD).

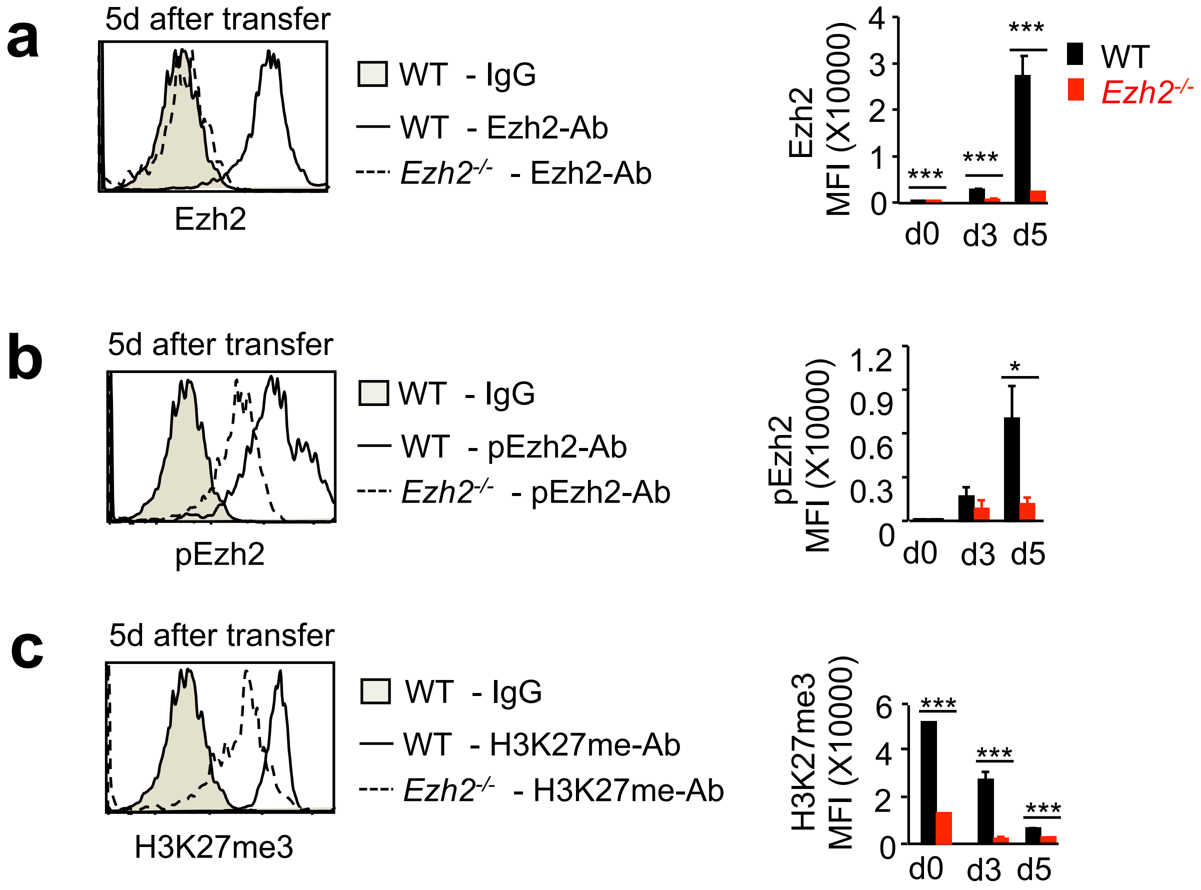

Supplementary Fig. 9. Ezh2 is progressively phosphorylated by Akt in activated CD8$^+$ T cells in vivo. WT (CD45.2$^+$Thy1.1$^+$) and *Ezh2*$^{-/-}$ (CD45.2$^+$Thy1.1$^-$) T$_N$ Pmel-1 cells were co-transferred into B6/SJL mice (CD45.1$^+$Thy1.1$^-$), followed with VVA-gp100 infection. Flow cytometric analysis was used to measure the expression of Ezh2 (**a**), H3K27me3 (**b**) and pEzh2 (**c**) in WT and *Ezh2*$^{-/-}$ T cells at 0d, 3d and 5d after transfer. Histograms (left) show the representative flow cytometric analysis results from donor T cells collected at 5d after transfer. Graphs (right) show the mean fluorescence intensity of tested molcules. Data are representative of two independent experiments with n=3 mice per group in each (mean ± SD).

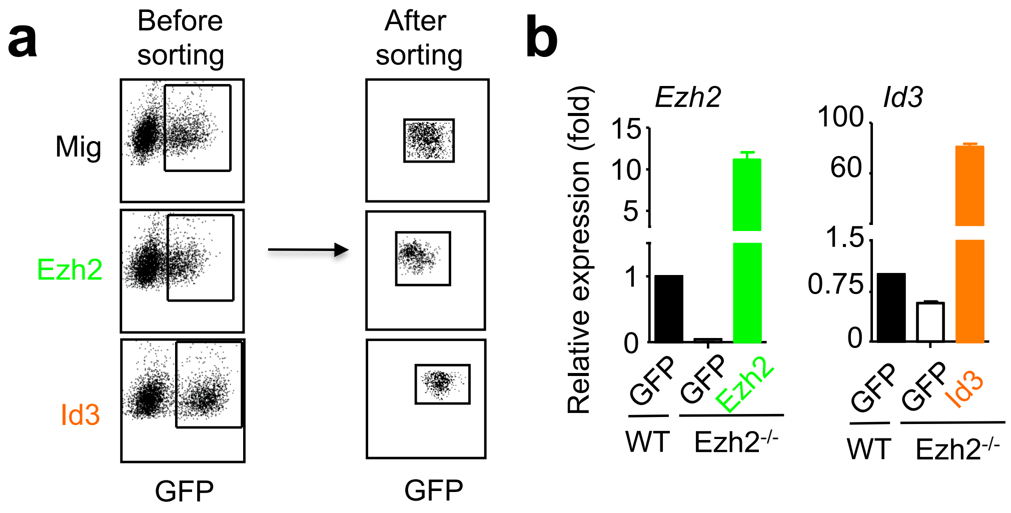

Supplementary Fig. 10. Retroviral introduction of Id3 into CD8^{+} T cells. *Ezh2*$^{-/-}$ Pmel-1 cells (Thy1.1^{+}) were stimulated with anti-CD3/CD28 Ab + IL-2 for 36 hrs, infected with MigR1 retrovirus encoding GFP, Ezh2, and Id3, respectively, and FACS sorted at 7d after culture based on the expression of GFP. (**a**) Plots show cells before and after sorting. (**b**) Real-time RT-PCR measurement of transduced genes. Data are representatives of two independent experiments.

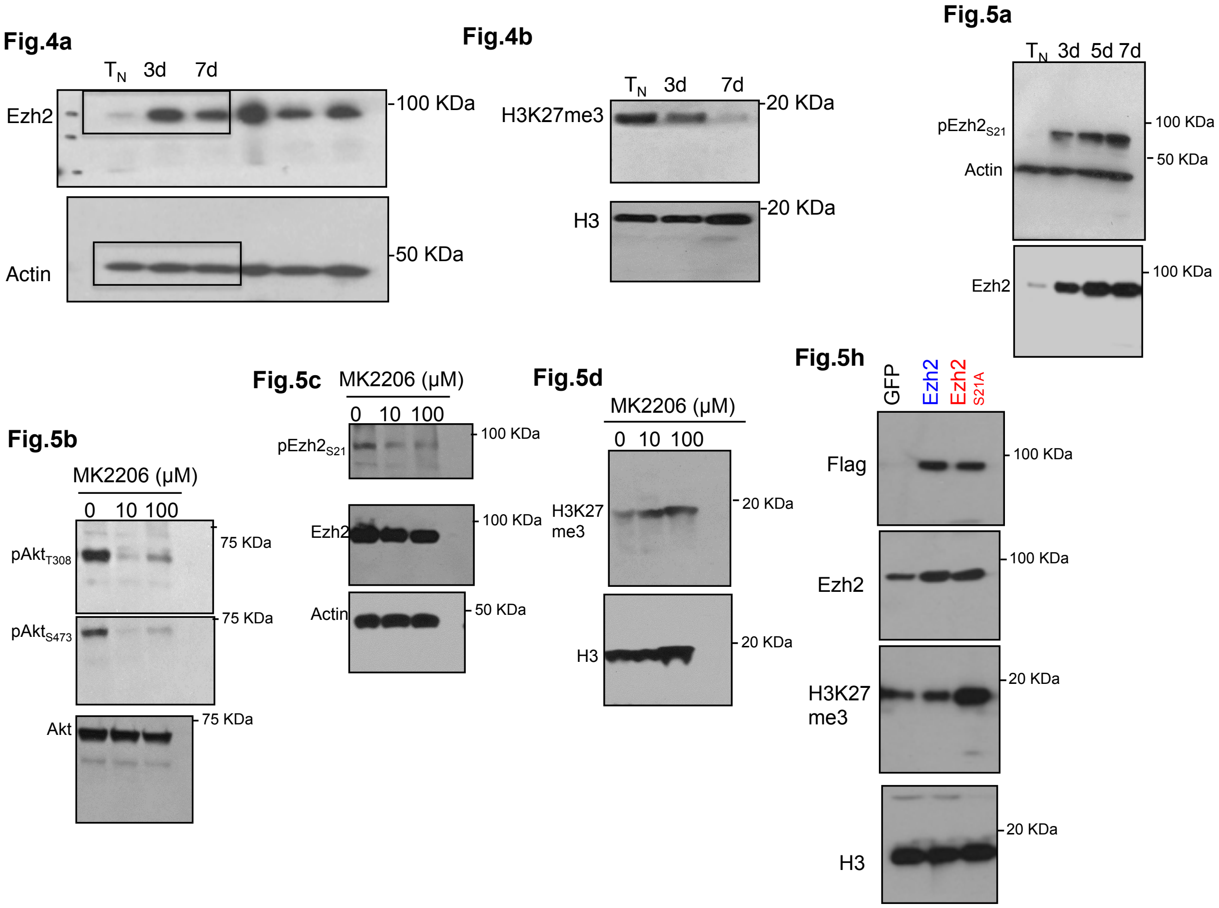

Supplementary Figure 11. Uncropped blots for western blot analysis.

Fig. 6b

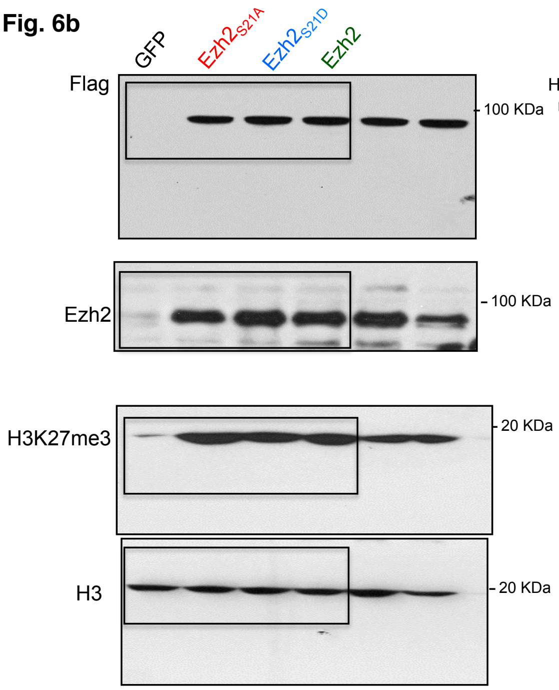

Fig. 8e

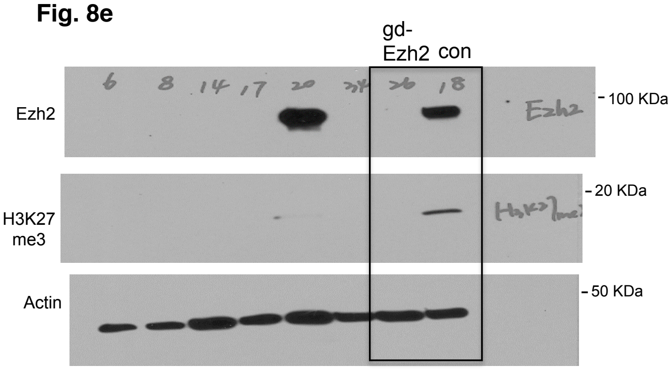

Supplementary Fig.6c

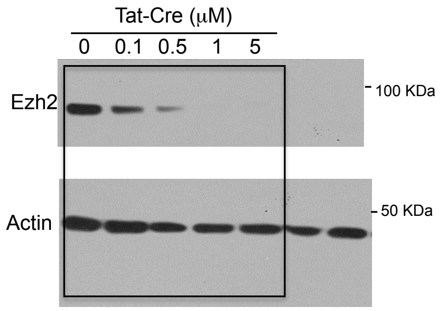

Supplementary Fig. 11 continued

Supplementary Fig.8a

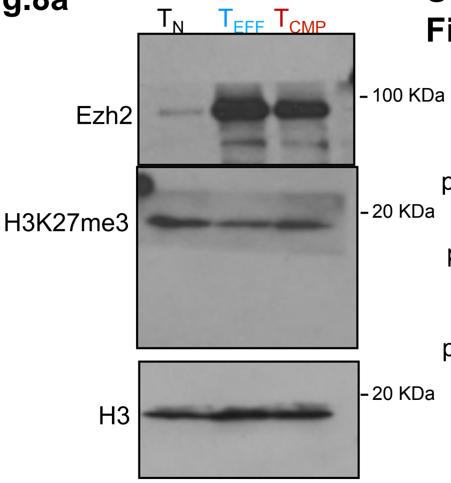

Supplementary Fig.8d

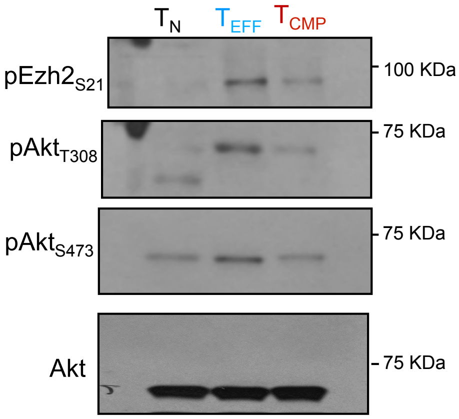

Supplementary Fig. 11 continued

Supplementary Table 1. Primers used for real-time RT-PCR

Gene name	Primer sequence	
18s	Forward	GCTGCTGGCACCAGACTT
	Reverse	CGGCTACCACATCCAAGG
Gapdh	Forward	AGGTCGGTGTGAACGGATTTG
	Reverse	TGTAGACCATGTAGTTGAGGTCA
Prdm1	Forward	ACAGGAGAGAAGCCACATGA
	Reverse	CGAAGGTGGGTCTTGAGATT
Eomes	Forward	GGCCCCTATGGCTCAAATTCC
	Reverse	CCTGCCCTGTTTGGTGAT
Bcl-6	Forward	AAAGGCCGGACACCAGTTTT
	Reverse	CCGGAGGCGATTAAGGTTGA
Tbx21	Forward	AGCAAGGACGGCGAATGTT
	Reverse	GGGTGGACATATAAGCGGTTC
Id3	Forward	AGCCTCTTGGACGACATGAA
	Reverse	AGCTCAGCTGTCTGGATCGG
Id2	Forward	ATGAAAGCCTTCAGTCCGGTG
	Reverse	AGCAGACTCATCGGGTCGT
Bim	Forward	GCCCGGCACCCATGAGTTGT
	Reverse	CGCCGCAGCTCCTGTGCAAT
Arf	Forward	GGATCTTGAGAAGAGGGCCG
	Reverse	GGAGAAGGTAGTGGGGTCCT
Dab2ip	Forward	AAATAGCGGCCCTGGAGGATGTTAG
	Reverse	GAGGGTGAGGAGAGGCGACTGC

Ifng	Forward	AGCTCTTCCTCATGGCTGTT
	Reverse	TTTGCCAGTTCCTCCAGATA
Kif2a	Forward	GCAGCTCGCGATGTCTTTTT
	Reverse	ACCTGCTGCTTCCCATCTTC
Kif4a	Forward	CCCTACTCCAAGTAGCCAGC
	Reverse	GGCAAGGTTTGGGCTTAGGT
Cdca5	Forward	AATCTGGCCGAGGACAACTC
	Reverse	TCGAACCGTGTGGACATCTG
Stk39	Forward	AGCTCTTCTCTGCTGGCTTG
	Reverse	CAGGAATCTCCGACCCATCG

Supplementary Table 2. Primers used for ChIP analysis

Gene name	locus	Primer sequence
Id3	-21.5k	AGGTAAGCTTTGGGTCGCAG
		GGGCCACAGCTGCAAGT
	-1.4k	CAGAGGTCCCATTGTCCTGT
		GAGAACAGGTGGGGTTTTGC
	-726	GGTCCATGCTTTTTCTTTCTCCGTGGAAAAGG
		GGGAAAAAATTAATTGCGGTGAAGCTGAGG
	-495	GAGGTCAGACGAGCAGCAAA
		CCAAGTTCTCTGAGGTCCCC
	-173	CGTCAGACCAGCCTAAGGAAG
		TTCAAAACAGACCGCCAAGG
	-120	CGCGCACTGTTTGCTGCTTTA
		CAGGCTACGTTCCGACAGG
	+3.1k	GGCCCGGACAAAAGCTTAGA
		CTGGTAGGCACGCCCATAAA
Eomes	-1.8k	TGGAAGGTCCTGCTGTTTGT
		GAAAACAAAGCGAGGGCACA
	-1.0k	CAGAGTGGGTCTGAACGGTG
		TTGAAGAGACCGGGGAAACG
	-679	CAAAAACATGCGGGACGGAG
		TTTGGCTCAGAGAACTCGGG
	-297	CATCTCGACCGGAAAATGCG
		GGGGAACACGTTTCGTTGAC

	-47	GAGAGGCGCAGGGAATCTTA
		GCCAACATCTTTGGTGGGGA
	+339	AAAAAGCGGTTCCTGTGTGC
		GCAAATCTAGGGGAGGGCTG
Tbx21	-601	CGTGAACGAGCTTTTGTGGG
		GATGCTTGTCCCTCTAGCCC
	-315	AGGCGTGAGAATGCTCAGAT
		AAACGGTTTCTCTCCCCCAG
	-245	CTTCCTGGGGGAGAGAAACC
		GGGAAAGAGTCAACCTGCCA
	-46	GGGACCCAAGGAGCTTCATA
		TGAAACTTCACTGGAGCGGG
	+131	CTCCCGCTCCAGTGAAGTTT
		AGAGACCCGAGGGTCCTTAG
Prdm1	-1.1k	CCAGGTATGTGCAGTGCTCC
		GGATTGGACAGCCTCTAGCC
	-598	TTTGACAGACGCCAGAGG
		CTTTCACAAGTTCGCATCGT
	-264	TTTCCATAATCTCAAAGACTCCTG
		GGGTTTGTCTGCTCAGAGGT
	-64	AGACGGATTGTAGCTGCCTT
		AAACAAGCAACTTCGGTGAA
	+8	GGAGTTTGTTGAGGCACAGA
		AAAGACCTTGCACTGTTCCA

Id2	-2.1k	GGGCTTGCTGTGACAGACTT
		CTGTTGGCACACTACCCAGT
	-750	TTCCAGTGTGCAAACCCCAC
		TCTCCCTCAGACCGCAGAG
	-324	TATAGCTAGCTCCGGGCACA
		ATTGTGCCCGCCTTATTCCA
	-109	TTCGCCTGGTATGATGGACG
		TTCAGAATCCCGGCAAGCG
	+115	AAAAACAGCCTGTCGGACCA
		GGCACCAGTTCCTTGAGCTT

Supplementary Table 3. Antibodies used for immunoblot and flow cytometry

Antibody	clone	Company	Catalog	Dilution	Validation Reference
Ezh2	11/EZH2	BD Bioscience	612667	1:1000	Supplier, refs online
Actin	13E5	Cell signaling	4970	1:3000	Supplier, refs online
H3K27me3	mAbcam 6002	Abcam	AB6002	1:3000	Supplier, refs online
H3		Cell signaling	9715	1:2500	Supplier, refs online
pEzh2S21		Bethyl lab	00388	1:2500	Science. 2012;338:1465
pAktT308	D25E6	Cell signaling	13038	1:1000	Supplier, refs online
PAktS473	D9E	Cell signaling	4060	1:2000	Supplier, refs online
Akt	C67E7	Cell signaling	4691	1:2000	Supplier, refs online
Id3	B72-1	BD Bioscience	556524	1:300	Supplier, refs online
Anti-FLAG-Peroxidase	M2	Sigma	A8592		Supplier, refs online
Anti-rabbit IgG-HRP		Cell signaling	7074	1:3000	Supplier, refs online
Anti-mouse IgG-HRP		Cell signaling	7076	1:3000	Supplier, refs online
APC anti-mouse CD44	IM7	BD Bioscience	559250	1:200	Supplier, refs online
APC/Cy7 anti-mouse CD8	53-6.7	Biolegend	100714	1:200	Supplier, refs online
PerCP/Cy5.5 Anti-rat/mouse Thy-1.1	OX-7	Biolegend	202515	1:200	Supplier, refs online
APC anti-mouse IFN-γ	XMG1.2	Biolegend	505810	1:100	Supplier, refs online
PE anti-mouse CD62L	MEL-14	BD Bioscience	553151	1:200	Supplier, refs online
PE anti-mouse CD122	TM-β1	BD Bioscience	553362	1:200	Supplier, refs online
PE anti-mouse CD127	SB/199	Biolegend	121111	1:200	Supplier, refs online
PE anti-mouse PD1	RMP1-30	eBioscience	12-9981-81	1:200	Supplier, refs online
APC anti-mouse KLRG	2F1	eBioscience	17-5893-81	1:200	Supplier, refs online
PE anti-mouse Id3	S30-778	BD Bioscience	564564	1:200	Supplier, refs online
PE Rat anti-mouse IgG1	A85-1	BD Bioscience	562027	1:200	Supplier, refs online
Ezh2 ChIP grade		Active motif	39901	5μl per ChIP	Supplier, refs online
H3K27me3 ChIP grade		Diagenode	PAb-069-050	1μg per ChIP	Supplier, refs online
IgG		Millipore	12-371	1μg per ChIP	Supplier, refs online
